# Fully automated waist circumference measurement on abdominal CT: Comparison with manual measurements and potential value for identifying overweight and obesity as an adjunct output of CT scan

**DOI:** 10.1371/journal.pone.0254704

**Published:** 2021-07-19

**Authors:** Ijin Joo, Min-Sun Kwak, Dae Hyun Park, Soon Ho Yoon

**Affiliations:** 1 Department of Radiology, Seoul National University Hospital and Seoul National Collage of Medicine, Seoul, Korea; 2 Department of Internal Medicine, Healthcare System Gangnam Center, Seoul National University Hospital, Seoul, Korea; 3 Department of Research and Development, MEDICALIP Co. Ltd., Seoul, Korea; Medical University of Vienna, AUSTRIA

## Abstract

**Objective:**

Waist circumference (WC) is a widely accepted anthropometric parameter of central obesity. We investigated a fully automated body segmentation algorithm for measuring WC on abdominal computed tomography (CT) in comparison to manual WC measurements (WC-manual) and evaluated the performance of CT-measured WC for identifying overweight/obesity.

**Materials and methods:**

This retrospective study included consecutive adults who underwent both abdominal CT scans and manual WC measurements at a health check-up between January 2013 and November 2019. Mid-waist WCs were automatically measured on noncontrast axial CT images using a deep learning-based body segmentation algorithm. The associations between CT-measured WC and WC-manual was assessed by Pearson correlation analysis and their agreement was assessed through Bland-Altman analysis. The performance of these WC measurements for identifying overweight/obesity (i.e., body mass index [BMI] ≥25 kg/m^2^) was evaluated using receiver operating characteristics (ROC) curve analysis.

**Results:**

Among 763 subjects whose abdominal CT scans were analyzed using a fully automated body segmentation algorithm, CT-measured WCs were successfully obtained in 757 adults (326 women; mean age, 54.3 years; 64 women and 182 men with overweight/obesity). CT-measured WC was strongly correlated with WC-manual (r = 0.919, *p* < 0.001), and showed a mean difference of 6.1 cm with limits of agreement between -1.8 cm and 14.0 cm in comparison to WC-manual. For identifying overweight/obesity, CT-measured WC showed excellent performance, with areas under the ROC curve (AUCs) of 0.960 (95% CI, 0.933–0.979) in women and 0.909 (95% CI, 0.878–0.935) in men, which were comparable to WC-manual (AUCs of 0.965 [95% CI, 0.938–0.982] and 0.916 [95% CI, 0.886–0.941]; *p* = 0.735 and 0.437, respectively).

**Conclusion:**

CT-measured WC using a fully automated body segmentation algorithm was closely correlated with manually-measured WC. While radiation issue may limit its general use, it can serve as an adjunctive output of abdominal CT scans to identify overweight/obesity.

## Introduction

Overweight and obesity is a global health problem with a rapidly increasing prevalence [1]. Body mass index (BMI), a value derived from body weight and height, is widely used to define overweight and obesity and is a risk factor for cardiovascular disease and type 2 diabetes [2, 3]. However, since BMI does not take into account the distribution of body fat, it has limitations in representing abdominal adiposity, which is an important aspect of body composition that would be linked to metabolic diseases [4].

Waist circumference (WC) is a simple but paramount anthropometric measurement that better reflects abdominal adiposity; therefore, WC is globally accepted as a screening tool for central obesity [5]. WC is not only closely correlated with BMI [6], but also provides independent or additive value to BMI for predicting obesity-related health risks [7–10]. Moreover, growing evidence suggests that WC is associated with all-cause [7–9] or cardiovascular mortality [10]. Nonetheless, since WC usually involves a manual procedure performed by medical personnel, it is prone to the risk of person-to-person spread of infectious diseases, such as coronavirus disease 2019, and is considered intimate in some cultures [11]. It would be beneficial if WC could be measured in routine clinical imaging without an additional close-contact examination. Several previous studies [12, 13] have tested the feasibility of obtaining WC measurements from CT images by drawing a region of interest or by step-by-step image analysis; however, those methods require time-consuming human intervention.

In recent years, deep learning-based algorithms have increasingly been investigated for automated segmentation of body composition from medical imaging [14, 15]. With these algorithms, three-dimensional (3D) information on body composition can be automatically extracted to obtain quantitative anthropometric parameters. Image slice selection based on the anatomical location and identification of the body perimeter in the selected slice could be automatically done, thereby enabling automatic WC measurements using CT images, which would provide a simple and practical imaging-driven parameter readily available in population-based cohorts. If CT-measured WC proves its value as an alternative or a surrogate to manually-measured WC, it could be used to evaluate patients with diseases for which manually-measured WC has already been found to be a useful prognostic indicator [7–10].

This study investigated a fully automated body segmentation algorithm for measuring WC on abdominal CT in comparison to manually-measured WC and evaluated the performance of CT-measured WC for identifying overweight and obesity.

## Materials and methods

This retrospective study was approved by the institutional review board of Seoul National University Hospital and Seoul National University Healthcare System Gangnam Center. The requirement for written informed consent was waived.

### Subjects

This study included subjects who had available manually-measured WC values (referred to as “WC-manual”), BMI information, and CT scans with full coverage of the entire abdomen taken for routine health check-ups at Seoul National University Healthcare System Gangnam Center from January 2013 to November 2019. Subjects were excluded if they were under 18 years old of age; or if the interval between the manual WC measurement or BMI and abdominal CT was more than 4 weeks. When a subject had a history of multiple visits during the study period, the most recent visit was chosen for the main analysis, and data from the previous visits were used for a subgroup analysis of longitudinal follow-up.

### Physical examinations

On the date of the visit, subjects completed a routine screening medical history, physical examination, and blood laboratory studies. The physical examination included height, body weight, and WC. Height was measured with a digital stadiometer to the nearest millimeter, and body weight was measured with a digital scale to the nearest 0.1 kg. Thereafter, BMI was calculated by dividing the body weight by height squared (kg/m^2^). Manual WC measurements were made using a tape measure to the nearest millimeter. In detail, WC was measured by placing an inelastic tape measure horizontally at the level of the mid‐point between the lower costal margin and the iliac crest at the end of a quiet expiration by well‐trained examiners according to the World Health Organization STEPwise approach to surveillance protocol [16]. During the measurements, subjects stood with their feet together and arms by their side.

### CT examination

Noncontrast phase CT images covering the entire abdomen were needed to extract CT-measured WCs in this study, so we collected abdominal CT scans using the kidney protocol, as that protocol for CT at our institution met the requirements. Subjects underwent abdominal CT examinations for various clinical indications, such as the characterization of ultrasound-detected renal lesions or follow-up of known renal lesions. CT examinations were performed using multi-detector row CT scanners (iCT 256: Philips Healthcare, Best, the Netherlands, n = 291; Sensation 16: Siemens Healthineers, Erlangen, Germany, n = 472). Noncontrast, corticomedullary, and nephrogenic phases were obtained before and after intravenous contrast administration (with delays of 30–40 s and 120 s, respectively), and their scan coverage ranged from above the diaphragmatic dome to below the pubic symphysis. Subjects were imaged during end-inspiration breath hold in the supine position while keeping their arms overhead. For noncontrast phase imaging, the CT scanning parameters were as follows: peak voltage, 120 kVp; tube current, 50 mA in the iCT 256 machine and a reference current-time product of 130 mAs with automatic tube current modulation in the Sensation 16 machine; field of view (FOV), approximately 30–40 cm for each axis depending on the patient’s size; slice thickness, 3 mm, and reconstruction interval, 3 mm.

### CT measurements of waist circumference

#### CT-measured WC

CT-measured WC referred to the waist perimeter, defined as the circumferential length of the outer margin of abdominal skin on axial CT images. An axial CT image taken at the midpoint of the z-axis level between the lowest rib margin and the highest iliac crest margin was chosen for WC measurement. This anatomical location was selected to reflect the principle of manual WC measurements used in this study. As detailed below, the z-axis level identification and WC measurement of a selected axis image were both performed in a fully automatic manner.

#### WC measurement using a fully automated body segmentation algorithm

**Fig 1:**
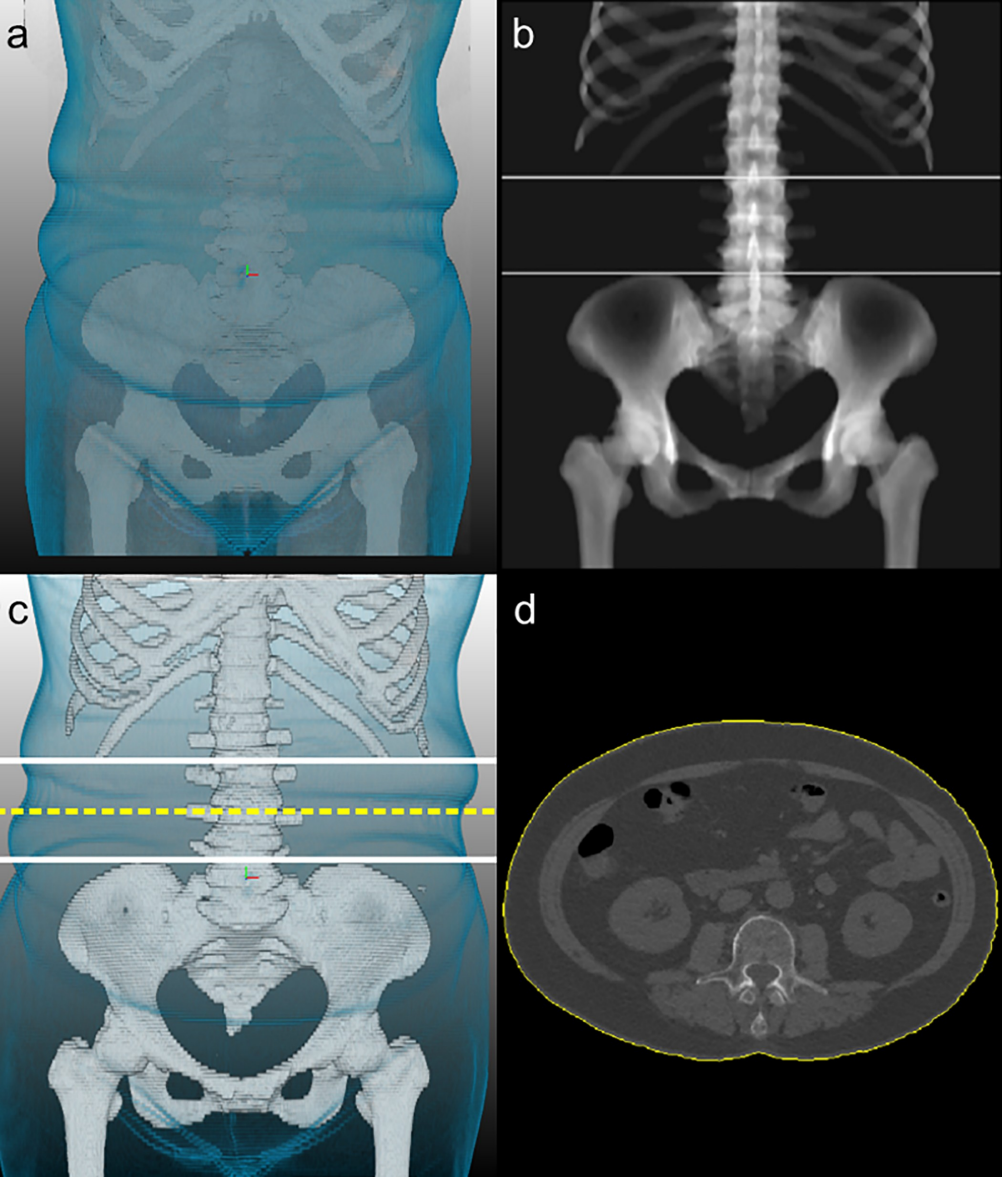
Example of a CT measurement of waist circumference using the fully automated body segmentation algorithm. (a) 3D display of skin and bone, (b) identification of the z-axis level of the lowest rib margin and the highest iliac crest margin, (c) determination of the midpoint z-axis level (dashed line) between the lowest rib margin and the highest iliac crest margin, (d) extracted body perimeter (yellow line) in the selected axial image based on 3D skin segmentation.

All WCs on CT were automatically measured using a commercially available software program (DeepCatch v1.0.0.0; MEDICALIP Co. Ltd., Seoul, Korea) (Fig 1). This program incorporates a body segmentation algorithm that works on either noncontrast or contrast CT scans. It uses a modified 3D U-Net to segment CT images into seven body components (skin, bone, muscle, abdominal visceral fat, subcutaneous fat, internal organs and vessels, and central nervous system) [17].

The principles of WC measurement on CT using this program were as follows: The z-axis levels of the lowest rib margin and the highest iliac crest margin were automatically determined based on the antero-posterior projection image of the 3D segmented bone mask. The axial CT image of the midpoint on the z-axis between those two levels (mid-waist) was identified on the projected image. Then, the WC value was extracted from the selected axial image of the 3D segmented skin mask by calculating the body perimeter connecting the outermost pixels of the skin. Of note, if there was any discontinuity in the skin mask on the image due to an insufficient FOV, we categorized the CT scan as “non-evaluable” for the WC measurement. This choice was made because an accurate WC measurement is technically impossible when the image does not show the whole circumference of the body, as can sometimes occur when the FOV of abdominal CT scans excludes the lateral portion of subcutaneous fat around the hip in the clinical setting.

#### Assessment of technical efficacy

The technical efficacy of WC measurements on CT was assessed by a visual assessment of automatically-generated body segmentation results by a radiologist (I.J. with 12 years of experience in abdominal imaging) who was blinded to the clinical parameters and CT-measured WC values of each subject. The radiologist reviewed the segmented WCs shown as color-coded lines on axial CT images on the segmentation software program. The CT measurement of WC for each subject was considered technically successful if the z-axis range of the waist (from the level of the lowest rib margin to the highest iliac crest margin) was correctly identified and all segmented WCs in the range fit the abdominal skin surface well.

### Statistical analysis

The association between CT-measured WC and WC-manual was assessed by Pearson correlation analysis, and their agreement was assessed using Bland-Altman analysis. In the subgroup of subjects with previous visits, the correlation and agreement between interval changes in CT-measured WC and WC-manual were assessed by Pearson correlation analysis and by Bland-Altman analysis, respectively. In addition, two-way consistency intra-class correlation coefficient (ICC) was calculated to evaluate the agreement between CT-measured WC and WC-manual.

The performance of CT-measured WC and WC-manual for identifying overweight/obesity (i.e., BMI ≥25 kg/m^2^) was evaluated using receiver operating characteristics (ROC) curve analysis, and the areas under the ROC curve (AUCs) for CT-measured WC and WC-manual were compared using the z-test. In addition, by using the maximal Youden index, the optimal cut-off value of CT-measured WC for overweight/obesity was estimated, and the corresponding sensitivity and specificity were calculated. The sensitivity and specificity of CT-measured WC were compared with those of WC-manual using the McNemar test.

All statistical analyses were performed using a commercially available software package (MedCalc, version 19.5.3, MedCalc Software, Ostend, Belgium), and *p-*values < 0.05 were regarded as indicating statistical significance.

## Results

### Subjects

Among 763 consecutive subjects whose abdominal CT scans were analyzed, CT-measured WC was successfully acquired in 757 subjects (326 women; mean age, 54.3 years; range, 19 to 82 years) resulting in technical efficacy of 99.2% (757/763), while not for the other 6 subjects (technical failure: n = 4, due to inaccurate bone segmentation potentially related to CT images with a low signal-to-noise ratio in 3 cases and inaccurate skin segmentation in 1 case; and non-evaluable WCs: n = 2, due to an insufficient FOV). The technical efficacy calculated in subjects with a sufficient FOV was 99.5% (757/761). The characteristics of the final study sample are described in Table 1. Their mean BMI was 23.8 kg/m^2^ (range, 16.0 to 39.0 kg/m^2^), and 32.5% (242/757; 64 women and 182 men) were categorized as overweight/obesity (BMI ≥25 kg/m^2^).

**Table 1:** Characteristics of the final study sample.

Characteristics		Total (n = 757)	Women (n = 326)	Men (n = 431)
Age	Mean ± SD (range)	54.3 ± 10.8 (19–82)	54.1 ±11.0 (22–82)	54.5 ± 10.6 (19–82)
BMI (kg/m^2^)	Mean ± SD (range)	23.8 ± 3.2 (16.0–39.0)	22.4 ± 3.2 (16.0–34.3)	24.7 ± 2.8 (17.9–39.0)
	<18.5 (underweight)	24 (3.2%)	21 (6.4%)	3 (0.7%)
	≥18.5 to <25 (normal)	487 (64.3%)	241 (73.9%)	246 (57.1%)
	≥25 to <30 (overweight)	217 (28.7%)	53 (16.3%)	164 (38.1%)
	≥30 (obesity)	29 (3.8%)	11 (3.4%)	18 (4.2%)
Manually-measured WC (cm)	mean ± SD (range)	85.3 ± 9.0 (63–121)	80.4 ± 8.5 (63–109)	88.9 ± 7.6 (66–121)

BMI = body mass index, SD = standard deviation, WC = waist circumference.

Of the final study sample, 41 subjects had available longitudinal data for a total of 49 previous visits (time interval: mean, 24 months; range, 4–67 months). The mean change in WC-manual between visits (the most recent value minus the previous value) was -1.4 cm [range, -11.0 to 6.0 cm].

### Relationship between CT-measured WC and manually-measured WC

**Fig 2:**
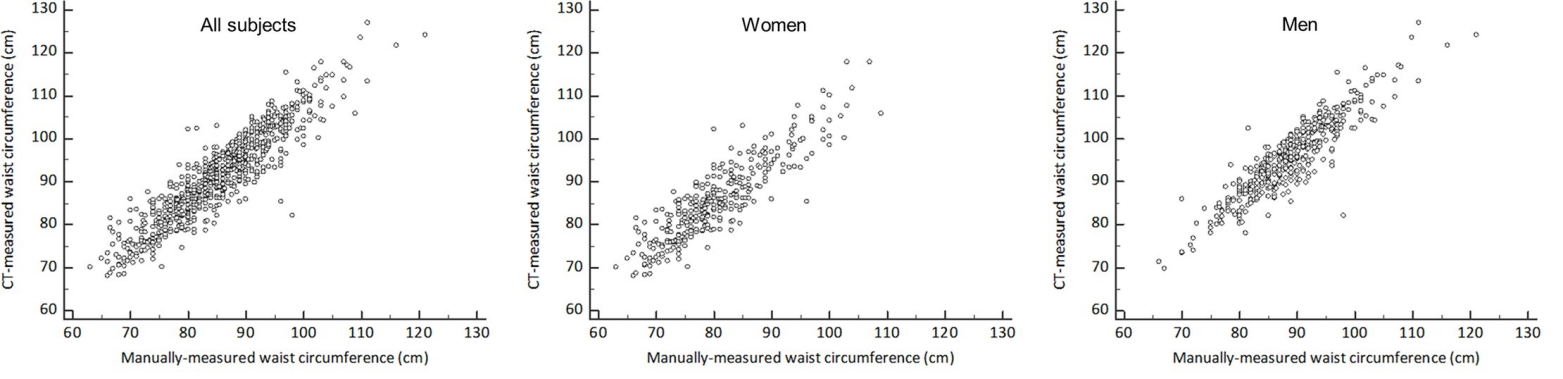
Scatter plots of CT-measured and manually-measured waist circumferences.

**Fig 3:**
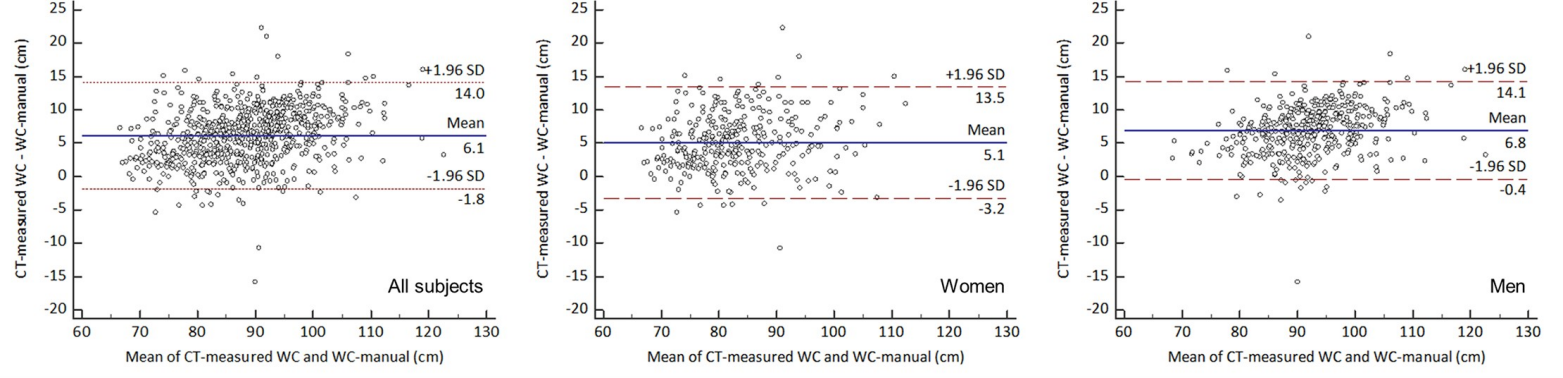
Bland-Altman plot of CT-measured versus manually-measured waist circumferences. WC = waist circumference, SD = standard deviation.

CT-measured WC was closely correlated with WC-manual (r = 0.919; 95% confidence interval [CI], 0.908–0.930], *p* < 0.001) (Fig 2). In addition, CT-measured WC and WC-manual showed a mean (± standard deviation [SD]) difference of 6.1 cm (± 4.0 cm), with limits of agreement (LoA) between -1.8 cm and +14.0 cm (Fig 3). ICC of CT-measured WC and WC-manual was 0.954 (95% CI, 0.947–0.960).

#### Subgroup analysis by sex

For both sexes, CT-measured WC and WC-manual showed strong positive correlations (women: r = 0.888; 95% CI, 0.863–0.909; *p* < 0.001; men: r = 0.904; 95% CI, 0.885–0.920; *p* < 0.001) (Fig 2). The mean (± SD) difference between CT-measured WC and WC-manual in women was 5.1 cm (± 4.3 cm) with LoA of -3.2 cm to +13.5 cm, while in men it was 6.8 cm (± 3.7 cm) with LoA of -0.4 cm to 1.4 cm (Fig 3). ICCs of two measurements were 0.939 (95% CI, 0.924–0.951) and 0.945 (95% CI, 0.933–0.954), respectively.

#### Subgroup analysis: A longitudinal follow-up

In the subgroup with serial data (49 pairs of recent and previous visits), the interval change of CT-measured WC and that of WC-manual showed a significant positive correlation (r = 0.308, *p* = 0.031) and a mean (± SD) difference of -0.3 cm (± 4.5 cm).

### Performance of CT-measured WC and manually-measured WC for identifying overweight and obesity

The performance of CT-measured WC and WC-manual to identify overweight/obesity (BMI ≥25 kg/m^2^) is summarized in Table 2. For each sex, CT-measured WC showed excellent performance, with AUCs of 0.960 in women and 0.909 in men, which were comparable to those of WC-manual (AUC of 0.965 for women and 0.916 for men; *p* = 0.735 and 0.437, respectively) (Table 2).

**Table 2:** Comparison of the performance of CT-measured and manually-measured weight circumference to identify overweight and obesity.

Performance of WC	CT-measured WC	Manually-measured WC	*p-*value
Women (n = 326)			
AUC (95% CI)	0.960 (0.933–0.979)	0.965 (0.938–0.982)	0.735
Sensitivity (%)	87.5 (56/64)	85.9 (55/64)	>0.999
Specificity (%)	92.7 (243/262)	89.7 (235/262)	0.308
Men (n = 431)			
AUC (95% CI)	0.909 (0.878–0.935)	0.916 (0.886–0.941)	0.437
Sensitivity (%)	83.5 (152/182)	81.9 (149/182)	0.690
Specificity (%)	83.9 (209/249)	83.9 (209/249)	>0.999

Note. The numbers in parentheses were used to calculate the percentages, unless otherwise specified. The cut-offs used to calculate sensitivity and specificity for identifying overweight/obesity were as follows: CT-measured WC, 91.7 cm in women and 96.7 cm in men; manually-measured WC, 85 cm in women and 90 cm in men. The *p-*values were obtained using the z-test or McNemar test. WC = waist circumference, AUC = area under the receiver operating characteristic curve, CI = confidence interval.

Based on ROC curve analysis, the optimal cut-off values for CT-measured WC for overweight/obesity in women and men were ≥91.7 cm and ≥96.7 cm, respectively, resulting in sensitivity and specificity values of 87.5% (56/64) and 92.7% (243/262); and 83.5% (152/182) and 83.9% (209/249), respectively. This performance was quite similar to that of WC-manual in both sexes, as determined by applying the WC cut-offs for Korean adults suggested by the Korean Society for the Study of Obesity (KSSO) (i.e., ≥85 cm in women and ≥90 cm in men, corresponding to a BMI ≥25 kg/m^2^) [18] (Table 2).

Moreover, using those cut-offs for overweight/obesity, CT-measured WC and WC-manual led to concordant results in 90.2% (294/326) of women and in 86.1% (371/431) of men (Table 3). When assessing subjects according to BMI categories, both the CT-measured WC cut-off and WC-manual cut-off for overweight/obesity did not result in any false positives in underweight subjects or any false negatives in obesity subjects. In both sexes, false positives in normal subjects and false negatives in overweight subjects were found at similar frequencies by CT-measured WC and WC-manual (Table 3).

**Table 3:** Patient categories according to CT-measured and manually-measured waist circumference for each body mass index category.

	Body mass index (kg/m^2^)			
Categories according to CT-measured and manually-measured WCs	<18.5 (underweight)	≥18.5 to <25 (normal)	≥25 to <30 (overweight)	≥30 (obesity)
Women	n = 21	n = 241	n = 53	n = 11
CT-measured WC <91.7 cm & WC-manual <85 cm	21 (100%)	206 (85.5%)	4 (7.5%)	0 (0%)
CT-measured WC <91.7 cm & WC-manual ≥85 cm*	0 (0%)	15 (6.2%)	4 (7.5%)	0 (0%)
CT-measured WC ≥91.7 cm & WC-manual <85 cm*	0 (0%)	8 (3.3%)	5 (9.4%)	0 (0%)
CT-measured WC ≥91.7 cm & WC-manual ≥85 cm	0 (0%)	12 (5.0%)	40 (75.5%)	11 (100%)
Men	n = 3	n = 246	n = 164	n = 18
CT-measured WC <96.7 cm & WC-manual <90 cm	3 (100%)	189 (76.8%)	19 (11.6%)	0 (0%)
CT-measured WC <96.7 cm & WC-manual ≥90 cm*	0 (0%)	17 (6.9%)	11 (6.7%)	0 (0%)
CT-measured WC ≥96.7 cm & WC-manual <90 cm*	0 (0%)	17 (6.9%)	14 (8.5%)	0 (0%)
CT-measured WC ≥96.7 cm & WC-manual ≥90 cm	0 (0%)	23 (9.3%)	120 (73.2%)	18 (100%)

Note.

*Discordant results between CT-measured WC and manually-measured WC for identifying overweight/obesity. WC = waist circumference, WC-manual = manually-measured WC.

## Discussion

Our study showed that using a fully automated body segmentation algorithm, WC could be successfully extracted from noncontrast abdominal CT images. Specifically, using 3D segmentation data of bone and skin from CT images, the body perimeter at the mid-waist level (= WC) was automatically calculated with a high technical success rate. The CT-measured WC was closely correlated with WC-manual (r = 0.919, *p* < 0.001; and ICC = 0.954) and provided excellent performance for identifying overweight/obesity (AUC, 0.960; 95% CI, 0.933–0.979 in women and 0.909; 95% CI, 0.878–0.935 in men), which were comparable to WC-manual in each sex. These results support the clinical applicability of automated CT-measured WC as a substitute for WC-manual. While radiation issue may limit the general use of CT to measure WC, given the high volume CT studies performed in a health check-up as well as disease monitoring, CT can provide a free additional information regarding WC, a well-established anthropometric parameter of central obesity and a diagnostic criterion for metabolic syndrome [5, 19].

In our study, despite the strong correlation of measured values, there existed an unignorable systematic difference between CT-measured WC and WC-manual (mean difference of 6.1 cm for all subjects). This discrepancy can be explained by differences in patient position (supine versus standing), breath control (expiration versus inspiration), and the measurement principle (perimeter including skin surface irregularities versus tape measurement). A previous study [20] also reported the difference in manually measured WCs according to the subject position (supine versus standing). The difference in these values indicates that CT-measured WC values are not directly interchangeable with WC-manual values. Hence, the cut-offs that have been validated for WC-manual may not be directly applied to CT-measured WC analyses. Indeed, the optimal cut-offs for CT-measured WC extracted from our study sample were 91.7 cm in women and 96.7 cm in men, which were substantially higher (by 6.7 cm in both women and men) than the KSSO criteria of WC-manual for Koreans (85 cm in women and 90 cm in men) [18]. Interestingly, when each cut-off value mentioned above was applied to CT-measured WC and WC-manual, the sensitivity and specificity of both methods were quite similar. This result might suggest that CT-measured WC and WC-manual could be used interchangeably if the measurements of the two methods are properly converted to each other.

In the subgroup analysis with longitudinal data, the interval change in CT-measured WC and WC-manual showed a significant positive correlation (r = 0.308, *p* = 0.031) with a minimal systematic difference (mean, -0.3 cm). This result may indicate the potential of CT-measured WC to be applied in a follow-up setting instead of WC-manual. Since a relative change in WC-manual has been proposed as a predictor of metabolic syndrome components [21] or mortality risk [22], serially-obtained CT-measured WC may also provide prognostic information in obese patients, although further validation is required.

There were several limitations in our study. First, this was a retrospective study of subjects who received routine health check-ups at a single center. To widen the generalizability of our study results, technical efficacy and measurement accuracy would need to be tested in different study populations such as cancer patients, and using various kinds of CT machines and scan protocols. Second, we evaluated the performance of WC for overweight/obesity, but not for obesity or severe obesity because only small numbers of subjects with obesity (less than 5% of the study sample) were included. Future studies are needed to determine the cut-offs for each obesity category in order for CT-measured WC to be utilized as a parameter providing detailed assessment and management guidance. Third, our study did not evaluate CT-measured WC as an indicator of metabolic conditions or as a predictor of clinical outcomes, although it indirectly showed its potential for such use.

In conclusion, CT-measured WC using a fully automated body segmentation algorithm was closely correlated with manually-measured WC, and can serve as an adjunct output of abdominal CT scans for overweight and obesity.

## Abbreviations

AUC: area under the receiver operating characteristics curve
BMI: body mass index
CI: confidence interval
FOV: field of view
ROC: receiver operating characteristics
WC: waist circumference
